# Australian Gay Men Describe the Details of Their HIV Infection Through a Cross-Sectional Web-Based Survey

**DOI:** 10.2196/jmir.5707

**Published:** 2016-09-23

**Authors:** Ian Down, Garrett Prestage, Jeanne Ellard, Kathy Triffitt, Graham Brown, Denton Callander

**Affiliations:** ^1^ Kirby Institute University of New South Wales Australia Sydney Australia; ^2^ Australian Research Centre in Sex, Health and Society La Trobe University Melbourne Australia; ^3^ Centre for Social Research in Health University of New South Wales Australia Sydney Australia

**Keywords:** HIV, transmission, sexual behavior, surveys and questionnaires

## Abstract

**Background:**

With emerging opportunities for preventing human immunodeficiency virus (HIV) transmission, it remains important to identify those at greatest risk of infection and to describe and understand the contexts in which transmissions occur. Some gay and bisexual men with recently diagnosed HIV infection are initially unable to identify high-risk behaviors that would explain their HIV infection. We explored whether Web-based data collection could assist them in identifying the circumstances of their infection.

**Objective:**

To assess the capacity of a Web-based survey to collect reliable self-report data on the event to which gay and bisexual men ascribe their HIV infection.

**Methods:**

The HIV Seroconversion Study included a Web-based survey of gay and bisexual men with recently diagnosed HIV infection in Australia. Participants were asked if they could identify and describe the event they believe led to their infection. Men were also asked about their sexual and other risk practices during the 6 months before their diagnosis.

**Results:**

Most (403/506, 79.6%) gay and bisexual men with newly diagnosed HIV infection were able to identify and describe the circumstances that likely led to their infection. Among those who were initially unable to identify possible exposure events, many could nonetheless provide sensible information that ostensibly explained their seroconversion. Free-text responses allowed men to provide more detailed and contextual information, whereas questions about the totality of their sexual behavior before diagnosis provided opportunities for men to describe their sexual risk behavior in general. Overall, 84.0% indicated having engaged in condomless anal intercourse before their HIV diagnosis, including 71.8% in the receptive position.

**Conclusions:**

This study demonstrates the effectiveness of using Internet-based technologies to capture sensitive information about the circumstances in which HIV infection occurs among gay and bisexual men. By providing a range of opportunities for relaying experience, this research reveals some of the complexity in how individuals come to understand and explain their HIV infection. These findings may assist in obtaining detailed sexual history in the clinical setting.

## Introduction

The diagnoses of human immunodeficiency virus (HIV) infection among gay and bisexual men continue to increase in Australia, and elsewhere [1,2]. Epidemiological studies have provided measures of per-contact probability of HIV transmission through various transmission modes [3,4]. Among gay and bisexual men, condomless anal intercourse presents the highest HIV risk exposure, particularly for those men who take the receptive position [4] and if their partner ejaculates inside the rectum [3]. Trends in condomless anal intercourse with casual partners have been found to be a strong predictor of trends in HIV infections among gay and bisexual men [5]. Furthermore, the presence of some sexually transmissible infections can facilitate HIV transmission [6].

Many gay and bisexual men have adapted their sexual behaviors to minimize the potential of HIV transmission [7-9]. The use of condoms during anal sex continues to be the primary method to reduce HIV transmission among gay and bisexual men [10-12]. In Australia, about a third of gay and bisexual men report no recent condomless anal intercourse (within the previous 6 months), whereas around 1 in 5 restrict condomless anal intercourse to seroconcordant regular partners [10]. However, recent behavioral surveillance data suggest that a sizeable proportion of men, more than a third, are engaging in condomless anal intercourse with casual partners [13].

While injecting drug use remains a common mode of transmission in many other settings [14], the practice has been attributed to fewer than 3% of all new HIV diagnoses in Australia over the past 5 years [2]. Although gay and bisexual men report injecting drug use at rates higher than their heterosexual peers [15], the actual proportion is nonetheless small and has been declining somewhat over the past decade [13]. Moreover, most Australian gay and bisexual men appear to adhere to safer injecting practices and are less likely than their heterosexual peers to share injecting equipment [16].

Studies of recent seroconverters have found HIV risk to be associated with sexual adventurism [17], the use of sex venues to meet partners [18,19], using illicit drugs while having sex [20,21], and the presence of sexually transmissible infections [6]. Data have also provided evidence of men acquiring HIV while attempting, and in the absence of, non–condom-based HIV risk reduction strategies [22]. In Australia, gay and bisexual men tend to have a fairly well-developed sense of the level of risk involved in particular sexual practices [23], and this perceived hierarchy of risks appears to broadly reflect what is known of relative risk [24].

The collection and analysis of reliable behavioral surveillance data is an essential part of an effective response to HIV. Self-report survey responses to measure sexual risk behaviors raise questions about reliability, with the potential effects of recall bias due to participants’ inability to accurately recall their sexual behavior, sensitivity about answering questions concerning sexual practices, and the unwillingness of some to acknowledge having engaged in risk behavior [25-27]. In clinic settings, when individuals provide this information to their doctor, what they describe may be no less subject to recall and social desirability bias. Indeed, there has been some suggestion that when collecting sensitive information, doing so in a clinic setting may inhibit full disclosure [27].

Such limitations may be even more pronounced when the data being collected concern a recent HIV diagnosis experience [28]. Previous research has indicated that between 20% and 36% of men are unable to identify a risk behavior that may have led to their infection [28,29]. One study found that men who were reluctant or unable to disclose information about condomless anal intercourse during surveys were later able to identify such events during face-to-face interviews [30].

Previous studies of HIV seroconversion in Australia have relied on clinical referral, involved clinic-based data collection, and were limited to sites in Sydney and Melbourne. Between 1993 and 2001, a total of 92 participants were interviewed (an average of 11.5 per year), whereas from 2003 to 2006, a total of 158 men were recruited (an average of almost 53 per year) [22,31]. These studies predated, or did not use, Web-based data collection, which may resolve these issues around recruitment and the sensitivity of data collection processes.

We sought to assess whether gay and bisexual men with recently diagnosed HIV infection could provide consistent and reliable information about their sexual risk behavior before their HIV diagnosis, using Web-based data collection techniques to capture information about possible exposures. Here, we report on sexual and other risk behavior data collected from these men, and compare these with known modes of transmission.

## Methods

### Eligibility Criteria

The HIV Seroconversion Study included a Web-based survey of people in Australia with recently diagnosed HIV infection. Eligibility criteria were being older than 18 years, living in Australia, and having been diagnosed HIV positive within the 2 years before enrollment. Ethics approval was obtained from the University of New South Wales and La Trobe University Human Research Ethics Committees.

### Recruitment

A convenience sample was obtained through a range of recruitment strategies: referrals from the staff of state AIDS Councils and organizations for people living with HIV, referrals from clinics (mostly sexual health services), or direct Web-based enrollment by individuals who have found a link to the survey posted on another website. The study was promoted on the websites of gay community organizations and in gay press. The only direct contact to recruit individuals took place either in a clinic setting or via a community-based organization. Eligible participants were directed to a dedicated study website, which provided information about the study, its purpose, and what participation involved. Participants were told that the survey was anonymous and would take around 20 minutes to complete. Participation was voluntary, and no incentives were offered. Those who chose to consent to participate were invited to begin the survey. As the survey was anonymous, participants’ consent is implied by their continuation. The study methods have been described in more detail elsewhere [32].

### Survey Questionnaire

The survey questionnaire was developed in consultation with community partners and was pilot-tested for usability and reliability. The questionnaire included demographic characteristics, details of participants’ HIV diagnosis, and their sexual and drug-taking behaviors both before and since their diagnosis. Respondents were able to navigate back to previous answers, should they wish to review their responses. Only key questions required a response, to ensure participants were only asked questions relevant to their circumstances.

Participants were asked if they could identify one or more events that they believed may have led to them acquiring HIV. Those who were not immediately able to identify an event were provided with information about potentially risky activities, such as anal intercourse without a condom, sharing a needle or other injecting equipment, or any other activity where they may have been exposed to someone's body fluids (such as blood or semen), and then asked again if they could identify an event that may have exposed them to HIV. Those who could identify their most likely highest-risk event were then asked questions about their sexual and other risk behavior at that event.

To capture additional detail, the behavioral survey questions were complemented by optional free-text questions, with men invited to provide further detail in their own words of any factors they believe contributed to their infection. These free-text responses were reviewed for sexual practices that may have facilitated the transmission of HIV and coded accordingly. Sex practices other than anal or oral intercourse were coded as semen-related (for example, ejaculating around the anus and then inserting a finger into the anus using the ejaculate as a lubricant) or blood-related (for example, fisting and sharing sex toys). An in-depth analysis of the free-text data collected through the Web-based survey is reported separately [33].

Finally, and separately, participants were asked about their sexual and injecting drug use behavior in the 6 months before diagnosis. They were asked specifically about anal intercourse, with and without condoms, according to their sexual position, with both regular and casual partners.

### Analysis

The quantitative data were analyzed with SPSS version 22 software. We report the range of behaviors that occurred at the highest-risk event as they were provided in participants’ survey responses—including those revealed in free-text responses—and other risk practices reported in the 6 months before their diagnosis.

## Results

### Study Participation

From December 2007 to March 2013, a total of 506 unique male respondents who reported that their HIV infection was due to homosexual contact had enrolled in the study. Most men (449/506, 88.7%) reported the year they received their diagnosis (Table 1). On average, over the time of these analyses, (449/5298) 8.47% of the eligible population enrolled in the study.

**Table 1:** Survey participant enrollment, by year of diagnosis and as proportion of eligible population.

Year	Survey respondents, n	Number of Australian diagnoses recorded, attributed to sex between men, N	Proportion of eligible population that participated in survey, %
2006	19	678	2.8
2007	53	702	7.5
2008	83	670	12.4
2009	82	691	11.9
2010	83	679	12.2
2011	69	806	8.6
2012	56	867	6.5
2013	4	205^a^	2.0
**Total**	449	5298	8.5

^a^Survey recruitment occurred only in the first 3 months of 2013, therefore we have provided one-fourth of the number of new diagnoses that occurred in 2013.

### Describing Risk Behaviors

Most of the 506 men (403/506, 79.6%) were able to identify one or more high-risk events that they believed may have led to their HIV infection without any prompting. Among those not immediately able to identify such an event, a further 51 men were able to recollect occasions where they may have been exposed to HIV after prompting. Of the 454 who could recall one or more high-risk events, almost half (202/454, 44.5%) identified one such event, whereas for the remaining men there was more than one occasion that may have resulted in their HIV infection (mean 7.32; standard deviation, SD, 12.06). These men were asked to select the event that was the most “risky” based on their own assessment using provided information about relative risk, with consideration to the time since their last HIV-negative test result, as well as a time when they may have experienced seroconversion-like symptoms. On the basis of this assessment, they were then asked to describe the event they felt was most likely to be their seroconversion event. A total of 403 men were able to describe a single highest-risk event. For these analyses, we included those 403 men, as well as an additional 62 men who provided free-text responses describing risk or responses to questions about their behaviors in the 6 months before diagnosis. Overall, (258/465), 55.5% were unable to describe the HIV status of their sex partner at the time of the highest-risk event, whereas (140/465) 30.1% believed him to be HIV negative and (67/465) 14.4% believed him to be HIV positive.

The 465 men included in these analyses ranged in age, at the time of their HIV diagnosis, from 16 to 73 years, with a mean of 34.5 (SD 9.5) years. Most men (306/465, 65.8%) completed the survey within 12 months of receiving their diagnosis, including (179/465) 38.5% who did so within 3 months of receiving their diagnosis. The vast majority of the men identified as gay (425/465, 91.4%), more than half (249/465, 53.6%) had some university education, and most had been born in Australia (364/465, 71.8%).

Details of the men’s HIV risk behaviors are presented in Table 2. In response to the direct survey questions about the sexual practices that occurred at their highest-risk event, most men reported anal intercourse at the time (384/465, 82.6%), with (322/465) 69.2% reporting condomless anal intercourse. Men most commonly (280/465, 60.2% of the men in these analyses) reported that they were the receptive partner, and (177/465) 38.1% reported that their partner ejaculated in their rectum. Among the men, (193/465) 41.5% were exclusively receptive during condomless anal intercourse, whereas (49/465) 10.5% were exclusively insertive. A small proportion of men (63/465, 13.5%) reported only condom-protected anal intercourse as their highest-risk event. Overall, 1 in 6 men (76/465, 16.3%) reported receptive oral intercourse with their partner ejaculating in their mouth. Of the 40 men (40/465, 8.6%) who reported injecting drug use as part of their highest-risk event, 5 indicated that they shared injecting equipment at the time.

In free-text responses, 94 men provided details about engaging in condomless anal intercourse at the highest-risk event. Few men (54/465, 11.6%) attributed their infection to sexual practices other than anal intercourse. Practices that may result in the transfer of blood between partners, such as fisting or sharing sex toys or douching equipment, were described by 21 men (21/465, 2.4%). The possibility of semen or pre-ejaculatory fluid entering the rectum was believed to be the mode of acquisition for 13 men, who described the brief, partial insertion of their partner’s penis into the anus without a condom (“dipping”) or their partner ejaculating around their anus and then inserting a finger into the anus. In their text responses, 1 in 10 men commented on abrasions on the skin or mucosal membranes in the mouth, anus, or penis that they believed might have facilitated infection. For 28 men, their responses were sufficient to reasonably imply that they had engaged in condomless anal intercourse; for example, when asked about what may have contributed to their acquiring HIV, one response was “my stupidity and horniness” and another was “too drunk, so didn’t take proper care.” These sorts of comments were often not accompanied by quantitative survey data or were accompanied by data that did not correspond.

In describing their sexual behavior during the 6 months before their HIV diagnosis, more than half of men (272/465, 58.5%) reported having engaged in condomless anal intercourse during that period. Men were more likely to report having been the receptive partner in condomless anal intercourse (241/465, 51.8%) than the insertive partner (191/465, 41.1%). Most men who reported receptive condomless anal intercourse had also done so to the point of ejaculation inside their rectum. There were 8 men who were unable to identify a highest-risk event and went on to describe occasions of condomless anal intercourse with casual partners in the 6 months before their diagnosis.

### Validating Responses

The 3 sources of information in the men’s responses were combined and reallocated according to their relative risk. These data are presented in Table 3 as a hierarchy of risk. We identified that (372/465) 84.0% of the men in this sample reported some condomless anal intercourse in the 6 months before their diagnosis. Almost three-fourths of the sample (334/465, 71.8%) reported receptive condomless anal intercourse, and (272/465) 58.5% of men had engaged in receptive condomless anal intercourse with ejaculation. Despite some discrepancies, and the provision of additional information in one set of questions versus other questions, there was broad consistency between the behaviors men reported through the survey responses and the descriptions provided in the free-text responses. Individual responses to the survey questions are compared with the individual free-text responses in Table 4.

**Table 2:** Risk behaviors reported at the highest-risk event and during the preceding 6 months.

Risk behaviors N=465		Direct response in survey (HRE^a^) n (%)	Text responses (about HRE) n (%)	Behavior in the preceding 6 months n (%)
**Anal intercourse**				
	Any anal intercourse	384 (82.6)	-	-
	Any receptive anal intercourse	317 (68.2)	-	-
	Any insertive anal intercourse	174 (37.4)	-	-
	Receptive anal intercourse only with a condom	39 (8.4)	-	-
	Condom slippage or breakage during receptive anal intercourse	25 (5.4)	10 (2.2)	-
	Insertive anal intercourse only with a condom	35 (7.5)	-	-
	Condom slippage or breakage during insertive anal intercourse	12 (2.6)	-	-
	Any condomless anal intercourse	322 (69.2)	-	272 (58.5)
	Any receptive condomless anal intercourse	280 (60.2)	-	241 (51.8)
	Receptive condomless anal intercourse with ejaculation	177 (38.1)	31 (6.7)	189 (40.6)
	Receptive condomless anal intercourse (ejaculation unspecified)	-	13 (2.8)	-
	Receptive condomless anal intercourse withdrawal	103 (22.1)	8 (1.7)	-
	Any insertive condomless anal intercourse	136 (29.2)	8 (1.7)	191 (41.1)
	Unspecified condomless anal intercourse	-	34 (7.3)	-
**Other sex activities identified as potentially leading to infection**				
	Receptive oral sex with ejaculation in mouth	76 (16.3)	5 (1.1)	-
	Receptive oral sex (ejaculation unspecified)	-	20 (4.3)	-
	Blood-related (eg, fisting, sharing toys)	-	21 (4.5)	-
	Semen-related (eg, ejaculate as lubricant, nudging or dipping)	-	13 (2.8)	-
	Wound, sore, or infection	109 (23.4)	50 (10.8)	-
	Risk implied although not specified	-	28 (6.0)	-
**Nonsexual risk**				
	Injecting drug use—without sharing equipment	36 (7.7)	-	35 (6.5)
	Injecting drug use—with shared equipment	4 (0.9)	2 (0.4)	7 (0.2)
	Tattoo, medical, or other blood to blood	-	5 (1.1)	-
**Could not identify likely risk**		59 (12.7)	-	-

^a^HRE: highest-risk event.

**Table 3:** Highest-risk behavior reported either at the highest-risk event or during the preceding 6 months.

Behavior N=465	n (%)
Receptive condomless sex with ejaculation	272 (58.5)
Receptive condomless sex (ejaculation unspecified)	7 (1.5)
Receptive condomless sex withdrawal	46 (9.9)
Condom slippage or breakage during receptive anal intercourse	9 (1.9)
Insertive condomless sex	54 (11.6)
Condom slippage or breakage during insertive anal intercourse	3 (0.6)
Semen-related sex act (eg, ejaculate as lubricant, nudging, or dipping)	5 (1.1)
Blood-related sex act (eg, fisting, sharing toys)	1 (0.2)
Receptive oral sex with ejaculation	6 (1.3)
Receptive oral sex (ejaculation unspecified)	3 (0.6)
Wound, sore, or infection	12 (2.6)
Injecting drug use—with shared equipment	2 (0.4)
Injecting drug use—without sharing equipment	4 (0.9)
Tattoo, medical, or other blood to blood	5 (1.1)
Only condom-protected anal intercourse	19 (4.1)
No clear evidence of risk	17 (3.7)

**Table 4:** Risk behaviors described in survey responses and free-text responses.

Risk behaviors	Anal intercourse, n							Other, n	Total, N
	RCLAI^a^ ejaculation	RCLAI withdrawal	Condom break RAI^b^	ICLAI^c^	Condom break IAI^d^	RAI with condom	IAI with condom		
RCLAI with ejaculation	20	3	0	2	0	1	1	4	31
RCLAI (ejaculation unspecified)	4	5	1	0	0	0	1	2	13
RCLAI withdrawal	3	3	0	1	0	1	0	0	8
Condom slippage/ breakage during RAI	0	2	5	0	2	1	0	0	10
ICLAI	0	1	0	6	1	0	0	0	8
Unspecified CLAI^e^	18	5	0	3	0	0	0	4	30
Semen-related sex act (eg, nudging)	1	3	0	0	0	2	1	6	13
Blood-related sex act (eg, fisting)	5	4	0	5	0	4	1	2	21
Receptive oral sex with ejaculation	0	0	0	1	0	0	1	3	5
Receptive oral sex (ejaculation unspecified)	3	1	0	0	0	2	2	5	13
Wound, sore, or infection	6	8	2	5	3	2	3	2	31
Injecting drug use-sharing	0	1	0	0	0	0	0	1	2
Tattoo, medical, or other blood to blood	0	0	0	0	0	0	0	5	5
Risk implied, although not specified	11	11	2	2	0	0	0	2	28
Only condom-protected anal intercourse	0	0	0	0	0	1	0	0	1
No clear evidence of risk	2	0	0	0	0	0	0	0	2
Unspecified anal intercourse	2	0	0	0	0	0	0	1	3
Unspecified RAI	0	1	0	0	0	0	0	0	1
No reference to risk in text response	102	55	2	24	2	9	2	44	240
**Total**	177	103	12	49	8	23	12	81	465

^a^RCLAI: receptive condomless anal intercourse.

^b^RAI: receptive anal intercourse.

^c^ICLAI: insertive condomless anal intercourse.

^d^IAI: insertive anal intercourse.

^e^CLAI: condomless anal intercourse.

## Discussion

### Principal Findings

We successfully employed Internet-based technologies to recruit a much larger and geographically extensive sample than obtained from earlier Australian seroconverter studies [22,31]. Aside from being a broader sample, the data we collected indicate that most men are both capable of and willing to identify and report their risk behavior in ways that make sense and appear reasonably reliable as a representation of their recollection and understanding of what occurred.

We were able to recruit on average nearly 100 men per year from across Australia, with fewer numbers recruited in later years as recruitment became less intensive because of reduced funding. This compares favorably with previous studies, which collected 15 and 53 participants per year in previous studies, all of those being from Sydney or Melbourne. The size of the sample recruited to this study is much larger than obtained from earlier studies, demonstrating that Internet-based studies can have much wider reach. During recruitment, a common resistance from some sites to refer patients to the study was concern about “burdening” people soon after diagnosis. That almost two-fifths of men completed the survey within 3 months of receiving their diagnosis suggests there is a willingness to participate in this kind of study, even while dealing with the impacts of their recent diagnosis.

The majority of men in this sample identified an occasion of receptive condomless anal intercourse as the mode through which they acquired HIV and, for most of those men, their partner ejaculated in their rectum at the time. This finding accords with what is known about the relative risk for HIV infection of specific practices, which suggests that most men in our sample appear capable of and willing to recall and report risk behavior. Some men identified multiple risk events, but most of them were able to identify what they believed was the highest-risk event. Additionally, some men reported having engaged in condomless anal intercourse during the 6 months before their diagnosis, some of which events may not have been identified by the men as potentially leading to their infection. Furthermore, a small number of men described practices that would otherwise be considered as safe, and these are explored in more detail through the qualitative data collected as part of this study [33]. A small number of men, however, were unable to identify a risk event to explain their infection, which has been noted in previous studies of people with recently diagnosed HIV infection [28,29]. In our study, recall was improved by the use of probing devices and free-text questions, and we were able to identify risk behaviors for many of these same men. In the end, most men reported receptive condomless anal intercourse in the period before their HIV diagnosis. This rate of receptive condomless anal intercourse, and particularly where ejaculation occurs in the rectum, was much higher than what has been found in surveys of Australian gay and bisexual men generally, particularly among HIV-negative gay and bisexual men, and even when compared with HIV-negative gay and bisexual men who engaged in condomless anal intercourse [10].

Although a small number of men reported injecting drug use during their highest-risk event, only a minority of those men reported sharing injecting equipment with others. This is consistent with what has previously been found among gay and bisexual men in Australia [16]. Nonetheless, after our detailed analysis of men's responses, it is clear that the only identifiable risk behavior for a small number of men was injecting drug use.

In the end, most men were both capable of and willing to identify and report their risk behavior in ways that make sense and appear reasonably reliable as a representation of their recollection and understanding of what led to their HIV infection. This study was able to identify rates of condomless anal intercourse at least as high as previous similar studies [28,29].

### Limitations

We excluded from our detailed analysis 41 men who could not identify a risk event or provide detail about their sexual behaviors in the 6 months before their diagnosis. We cannot know why they did not complete these sections of the survey, nor can we account for them. Our descriptions here, though, are of men who were at least able to recognize, or describe, something that had happened that put them at risk of infection. Although we did not identify any duplicate entries, it is possible that individuals may have entered their responses more than once.

We cannot determine from these data that the events men believed led to their infection were the actual source of their infection. More than half of the men reported multiple possible events.

Over the last decade, there have been significant changes in how gay men connect with each other [34] and their sense of community [35]. These changes have run parallel to changes in HIV prevention, with focus on emerging biomedical approaches [36-38]. It is therefore understandable that for some men there may be uncertainty about the current relative risk of particular practices [39]. Definitions of what is safe and what is not safe may differ between men and may be changing for individual men. In addition, the ways that the relative risk of specific sex practices are assessed within gay communities appear to be changing, further adding to the possibility of confusion for individual men [40].

Although self-report data on sexual risk behavior are subject to limitations, anonymous Web-based surveys that present culturally appropriate questions have been found to produce reliable data [25]. There are particular challenges to accurately measuring sexual behavior, given that “the object under consideration—sexual practice and its change—is fluid, embedded in specific social formations, and involves the negotiation of meaning” [41,42]. Surveys are discursive and iterative in that they can often imply narrow meanings and interpretation in ways that do not always reflect what was intended, and may miss important mediating factors or contextual influences. Some of the men who did not provide responses to the set survey questions about the activity that occurred at the time they became infected did utilize the free-text component of the survey to describe and explore other events that may have led to infection, including less common explanations for their transmission, which is the subject of a separate analysis [33].

Given this interpretive variability, even well-developed survey questions can lead to confusion between the researchers’ intention and the respondents’ interpretation. With opportunity, many men can and will offer further contextual explanations in relation to what they believe led to their HIV infection, demonstrating the value of including free-text opportunities in survey questionnaires where possible. This contextual detail suggests that some men require an opportunity to articulate their story in greater detail, including the reasons they found themselves in a particular situation. Motivation for participants’ sexual behavior was not asked in the survey questionnaire.

### Conclusions

These findings suggest that seroconverter studies could take advantage of Internet-based technologies to obtain a broad sample of individuals with recently diagnosed HIV infection. Our data demonstrate a willingness for people with recently diagnosed HIV infection to engage in such research and that this method can collect, what appear to be, reasonably reliable data. Individuals with newly diagnosed HIV infection are generally able to identify and describe the likely circumstances that led to their infection. However, for some individuals, this may not always be so straightforward. Providing multiple and accessible methods for people to describe how they believe they were infected may allow some people to explain what they believe happened, even if they could not do so using one particular method. Providing diverse opportunities for relaying experience can sometimes elicit more detailed and contextual information. Given that identifying the circumstances that led to a person's HIV infection is a high priority, providing opportunities for alternative ways of describing the details of what occurred, within survey instruments and through qualitative methods such as narrative interviews, strengthens the evidence base for more effective HIV prevention and support work. In the changing environment, where the circumstances of seroconversion are likely to be more complex, the capacity to reliably collect sensitive information remains important. In the context of limited resources, this appears to be an efficient and reliable method.

In addition to clinic-based data collection, Internet-based technologies should be employed to obtain detailed data about the circumstances in which individuals believe they were infected with HIV.

## Abbreviations

HIV: human immunodeficiency virus
SD: standard deviation

## References

[1] Beyrer C, Baral SD, van Griensven F, Goodreau SM, Chariyalertsak S, Wirtz AL, Brookmeyer R (2012). Global epidemiology of HIV infection in men who have sex with men. Lancet.

[2] (2015). The Kirby Institute.

[3] Jin F, Jansson J, Law M, Prestage GP, Zablotska I, Imrie JC, Kippax SC, Kaldor JM, Grulich AE, Wilson DP (2010). Per-contact probability of HIV transmission in homosexual men in Sydney in the era of HAART. AIDS.

[4] Vittinghoff E, Douglas J, Judson F, McKirnan D, MacQueen K, Buchbinder SP (1999). Per-contact risk of human immunodeficiency virus transmission between male sexual partners. Am J Epidemiol.

[5] Zablotska IB, Prestage G, Middleton M, Wilson D, Grulich AE (2010). Contemporary HIV diagnoses trends in Australia can be predicted by trends in unprotected anal intercourse among gay men. AIDS.

[6] Jin F, Prestage GP, Imrie J, Kippax SC, Donovan B, Templeton DJ, Cunningham A, Mindel A, Cunningham PH, Kaldor JM, Grulich AE (2010). Anal sexually transmitted infections and risk of HIV infection in homosexual men. J Acquir Immune Defic Syndr.

[7] Dubois-Arber F, Jeannin A, Lociciro S, Balthasar H (2012). Risk reduction practices in men who have sex with men in Switzerland: serosorting, strategic positioning, and withdrawal before ejaculation. Arch Sex Behav.

[8] Prestage G, Van De Ven P, Grulich A, Kippax S, McInnes D, Hendry O (2001). Gay men's casual sex encounters: discussing HIV and using condoms. AIDS Care.

[9] Van de Ven P, Kippax S, Crawford J, Rawstorne P, Prestage G, Grulich A, Murphy D (2002). In a minority of gay men, sexual risk practice indicates strategic positioning for perceived risk reduction rather than unbridled sex. AIDS Care.

[10] Mao L, Kippax SC, Holt M, Prestage GP, Zablotska IB, de Wit JBF (2011). Rates of condom and non-condom-based anal intercourse practices among homosexually active men in Australia: deliberate HIV risk reduction?. Sex Transm Infect.

[11] Saxton PJW, Dickson NP, Hughes AJ (2014). Location-based HIV behavioural surveillance among MSM in Auckland, New Zealand 2002-2011: condom use stable and more HIV testing. Sex Transm Infect.

[12] Pines HA, Gorbach PM, Weiss RE, Shoptaw S, Landovitz RJ, Javanbakht M, Ostrow DG, Stall RD, Plankey M (2014). Sexual risk trajectories among MSM in the United States: implications for pre-exposure prophylaxis delivery. J Acquir Immune Defic Syndr.

[13] de Wit J, Mao L, Adam P, Treloar C (2014). CSRH.

[14] Degenhardt L, Mathers BM, Wirtz AL, Wolfe D, Kamarulzaman A, Carrieri MP, Strathdee SA, Malinowska-Sempruch K, Kazatchkine M, Beyrer C (2014). What has been achieved in HIV prevention, treatment and care for people who inject drugs, 2010-2012? A review of the six highest burden countries. Int J Drug Policy.

[15] Lea T, Mao L, Bath N, Prestage G, Zablotska I, de Wit J, Holt M (2013). Injecting drug use among gay and bisexual men in Sydney: prevalence and associations with sexual risk practices and HIV and hepatitis C infection. AIDS Behav.

[16] Grulich AE, de Visser RO, Smith AM, Rissel CE, Richters J (2003). Sex in Australia: injecting and sexual risk behaviour in a representative sample of adults. Aust N Z J Public Health.

[17] Kippax S, Campbell D, Van de Ven P, Crawford J, Prestage G, Knox S, Culpin A, Kaldor J, Kinder P (1998). Cultures of sexual adventurism as markers of HIV seroconversion: a case control study in a cohort of Sydney gay men. AIDS Care.

[18] Thiede H, Jenkins RA, Carey JW, Hutcheson R, Thomas KK, Stall RD, White E, Allen I, Mejia R, Golden MR (2009). Determinants of recent HIV infection among Seattle-area men who have sex with men. Am J Public Health.

[19] Silva AP, Greco M, Fausto MA, Greco DB, Carneiro M (2014). Risk factors associated with HIV infection among male homosexuals and bisexuals followed in an open cohort study: Project Horizonte, Brazil (1994-2010). PLoS One.

[20] Volk JE, Prestage G, Jin F, Kaldor J, Ellard J, Kippax S, Grulich AE (2006). Risk factors for HIV seroconversion in homosexual men in Australia. Sex Health.

[21] Plankey MW, Ostrow DG, Stall R, Cox C, Li X, Peck JA, Jacobson LP (2007). The relationship between methamphetamine and popper use and risk of HIV seroconversion in the multicenter AIDS cohort study. J Acquir Immune Defic Syndr.

[22] Jin F, Prestage GP, Ellard J, Kippax SC, Kaldor JM, Grulich AE (2007). How homosexual men believe they became infected with HIV: the role of risk-reduction behaviors. J Acquir Immune Defic Syndr.

[23] Mao L, Adam P, Kippax S, Holt M, Prestage G, Calmette Y, Zablotska I, de Wit J (2013). HIV-negative gay men's perceived HIV risk hierarchy: imaginary or real?. AIDS Behav.

[24] Jin F, Crawford J, Prestage GP, Zablotska I, Imrie J, Kippax SC, Kaldor JM, Grulich AE (2009). Unprotected anal intercourse, risk reduction behaviours, and subsequent HIV infection in a cohort of homosexual men. AIDS.

[25] Fenton KA, Johnson AM, McManus S, Erens B (2001). Measuring sexual behaviour: methodological challenges in survey research. Sex Transm Infect.

[26] Hoff CC, Faigeles B, Wolitski RJ, Purcell DW, Gomez C, Parsons JT (2004). Sexual risk of HIV transmission is missed by traditional methods of data collection. AIDS.

[27] Catania JA (1999). A framework for conceptualizing reporting bias and its antecedents in interviews assessing human sexuality. Journal of Sex Research.

[28] Moskowitz DA (2010). Claiming HIV infection from improbable modes as a possible coping strategy. J Appl Soc Psychol.

[29] Richters J, Grulich A, Ellard J, Hendry O, Kippax S (2003). HIV transmission among gay men through oral sex and other uncommon routes: case series of HIV seroconverters, Sydney. AIDS.

[30] Keet IP, Albrecht van Lent N, Sandfort TG, Coutinho RA, van Griensven GJ (1992). Orogenital sex and the transmission of HIV among homosexual men. AIDS.

[31] Kippax S, Slavin S, Ellard J, Hendry O, Richters J, Grulich A, Kaldor J (2003). Seroconversion in context. AIDS Care.

[32] Down I, Ellard J, Triffitt K, Brown G, Prestage G (2015). Factors associated with recent previous HIV testing among a sample of recently HIV-diagnosed gay men in Australia: a cross-sectional study. Sex Transm Infect.

[33] Callander D, Prestage G, Ellard J, Triffitt K, Brown G, Down I (2016). The Road Less Travelled: Exploring Gay and Bisexual Men's Explanations of 'Uncommon' Routes of HIV Transmission. AIDS Behav.

[34] Prestage G, Bavinton B, Grierson J, Down I, Keen P, Bradley J, Duncan D (2015). Online dating among Australian gay and bisexual men: romance or hooking up?. AIDS Behav.

[35] Zablotska IB, Holt M, Prestage G (2012). Changes in gay men's participation in gay community life: implications for HIV surveillance and research. AIDS Behav.

[36] Cohen MS, Chen YQ, McCauley M, Gamble T, Hosseinipour MC, Kumarasamy N, Hakim JG, Kumwenda J, Grinsztejn B, Pilotto JHS, Godbole SV, Mehendale S, Chariyalertsak S, Santos BR, Mayer KH, Hoffman IF, Eshleman SH, Piwowar-Manning E, Wang L, Makhema J, Mills LA, de BG, Sanne I, Eron J, Gallant J, Havlir D, Swindells S, Ribaudo H, Elharrar V, Burns D, Taha TE, Nielsen-Saines K, Celentano D, Essex M, Fleming TR, HPTN 052 Study Team (2011). Prevention of HIV-1 infection with early antiretroviral therapy. N Engl J Med.

[37] Grant RM, Lama JR, Anderson PL, McMahan V, Liu AY, Vargas L, Goicochea P, Casapía Martín, Guanira-Carranza JV, Ramirez-Cardich ME, Montoya-Herrera O, Fernández Telmo, Veloso VG, Buchbinder SP, Chariyalertsak S, Schechter M, Bekker L, Mayer KH, Kallás Esper Georges, Amico KR, Mulligan K, Bushman LR, Hance RJ, Ganoza C, Defechereux P, Postle B, Wang F, McConnell JJ, Zheng J, Lee J, Rooney JF, Jaffe HS, Martinez AI, Burns DN, Glidden DV, iPrEx Study Team (2010). Preexposure chemoprophylaxis for HIV prevention in men who have sex with men. N Engl J Med.

[38] Conway DP, Guy R, Davies SC, Couldwell DL, McNulty A, Smith DE, Keen P, Cunningham P, Holt M, Sydney Rapid HIV Test Study (2015). Rapid HIV Testing Is Highly Acceptable and Preferred among High-Risk Gay And Bisexual Men after Implementation in Sydney Sexual Health Clinics. PLoS One.

[39] Prestage G, Brown G, Down IA, Jin F, Hurley M (2013). “It's hard to know what is a risky or not a risky decision”: gay men's beliefs about risk during sex. AIDS Behav.

[40] McKechnie ML, Bavinton BR, Zablotska IB (2013). Understanding of norms regarding sexual practices among gay men: literature review. AIDS Behav.

[41] Race K (2014). The difference practice makes: Evidence, articulation, and affect in HIV prevention. AIDS Educ Prev.

[42] Kippax S, Stephenson N (2005). Meaningful evaluation of sex and relationship education. Sex Education.

